# Treatment of Complicated Hepatic Alveolar Echinococcosis Disease With Suspicious Lymph Node Remote Metastasis Near the Inferior Vena Cava-Abdominal Aorta: A Case Report and Literature Review

**DOI:** 10.3389/fonc.2022.849047

**Published:** 2022-03-24

**Authors:** Xiaolei Xu, Cancan Gao, Xinye Qian, Hong'en Liu, Zhan Wang, Hu Zhou, Ying Zhou, Haijiu Wang, Lizhao Hou, Shaoshuai He, Xiaobin Feng, Haining Fan

**Affiliations:** ^1^ Department of Hepatobiliary and Pancreatic Surgery, Qinghai University Affiliated Hospital, Xining, China; ^2^ Center of Hepatobiliary Pancreatic Disease, Beijing Tsinghua Changgung Hospital, Beijing, China; ^3^ The Research Key Laboratory for Echinococcosis of Qinghai Province, Xining, China

**Keywords:** hepatic alveolar echinococcosis (HAE), surgery, pathological, metastasis, lymph nodes

## Abstract

Echinococcosis is a human-animal parasitic disease caused by Echinococcosis tapeworm larvae in humans. From a global perspective, it is mainly prevalent in the mid-high latitudes of the Northern Hemisphere, and it is a widespread infectious disease. Its form, host and release areas are slightly different. In clinical practice, *Echinococcus granulosus* (hepatic cystic echinococcosis) is the most common. Its growth mode is swelling growth and its metastasis is more common in implanted metastasis; However, hepatic alveolar echinococcosis (HAE) is rare. It has been reported that HAE can metastasize through the blood or lymph nodes, and its invasive growth pattern is known as “carcinoma”. At this time, it may be accompanied by invasion of the portal vein and inferior vena cava(IVC)or metastasis to distant organs outside the liver (such as lung, brain, lymph nodes). Most patients are in the middle or late stages, making treatment complicated. World Health Organization guidelines recommend radical resection of HAE; However, there is no consensus on lymph node dissection. To date, there have been no reports of cases of HAE accompanied by inferior vena cava-para-abdominal aortic suspected lymph node metastasis and infection. This article reports a clinical case of a complex HAE treated by the surgical method of “middle liver resection + abdominal enlarged lymph node resection + inferior vena cava repair”, and histological examination was performed to illustrate the differences in microscopic pathology of alveolar echinococcosis invading the liver and lymph nodes at different magnifications. This article reviews the relevant literature on HAE and derives the latest treatment methods for HAE to provide a reference for future clinical cases of similar alveolar echinococcosis and maximize the benefits of patients.

## Introduction

Hepatic alveolar echinococcosis (HAE) is a zoonotic parasitic disease caused by echinococcosis larvae infecting the host that is mainly prevalent in the Northern Hemisphere and poses a great risk to human health (1–3). After echinococcus eggs are accidentally ingested, they enter the duodenum, and more than 70% enter the portal system and stay in the liver, where they develop into cysts and form hepatic echinococcus. Hepatic echinococcosis can affect the entire liver, destroying its anatomical structure and impair its function. Drug therapy for hydatid disease (albendazole) has a long treatment cycle, and there is currently a lack of specific drugs. If effective intervention is not carried out, the disease will gradually aggravate and worsen. Most patients can occur obstructive jaundice when the lesion enlarges and erodes the biliary tract. If the liquefied cavity is infected with secondary infection, it can form an abscess. The large lesion erodes most of the liver and can be combined with portal hypertension, liver function decompensation, and eventually death due to liver failure, biliary system infection, and metastasis of the lung, brain and other organs (4). There have been a large number of literature reports on imaging studies of HAE and extrahepatic blood metastasis at home and abroad (5), but currently, there are few reports on the imaging characteristics of lymph node metastasis. The metastatic mechanism of HAE is still unclear (6). In particular, the enlarged lymph nodes are located between the inferior vena cava and adjacent to the abdominal aorta, and close to the kidneys and vertebral bodies; However, common enlarged lymph node are mostly located next to the hepatoduodenal ligament clinically. There are no reported cases of suspicious lymph node metastases of echinococcosis with such a specific location of the lesion, which makes treatment more difficult. This paper reports a case of HAE with suspicious lymph node metastasis who underwent “middle liver resection + enlarged lymphadenectomy + inferior vena cava repair”. During the operation, strictly following the principle of being tumour-free, most lesions are removed away from the important bile ducts and blood vessels that need to be preserved, the remaining lesions are finely stripped, and then the inferior vena cava-para-aortic lymph nodes are finely stripped to reach skeletonization, which is also in line with the concept of precise hepatectomy (7), preventing postoperative echinococcosis recurrence and improving the quality of life.

## Case Report

**Figure 1:**
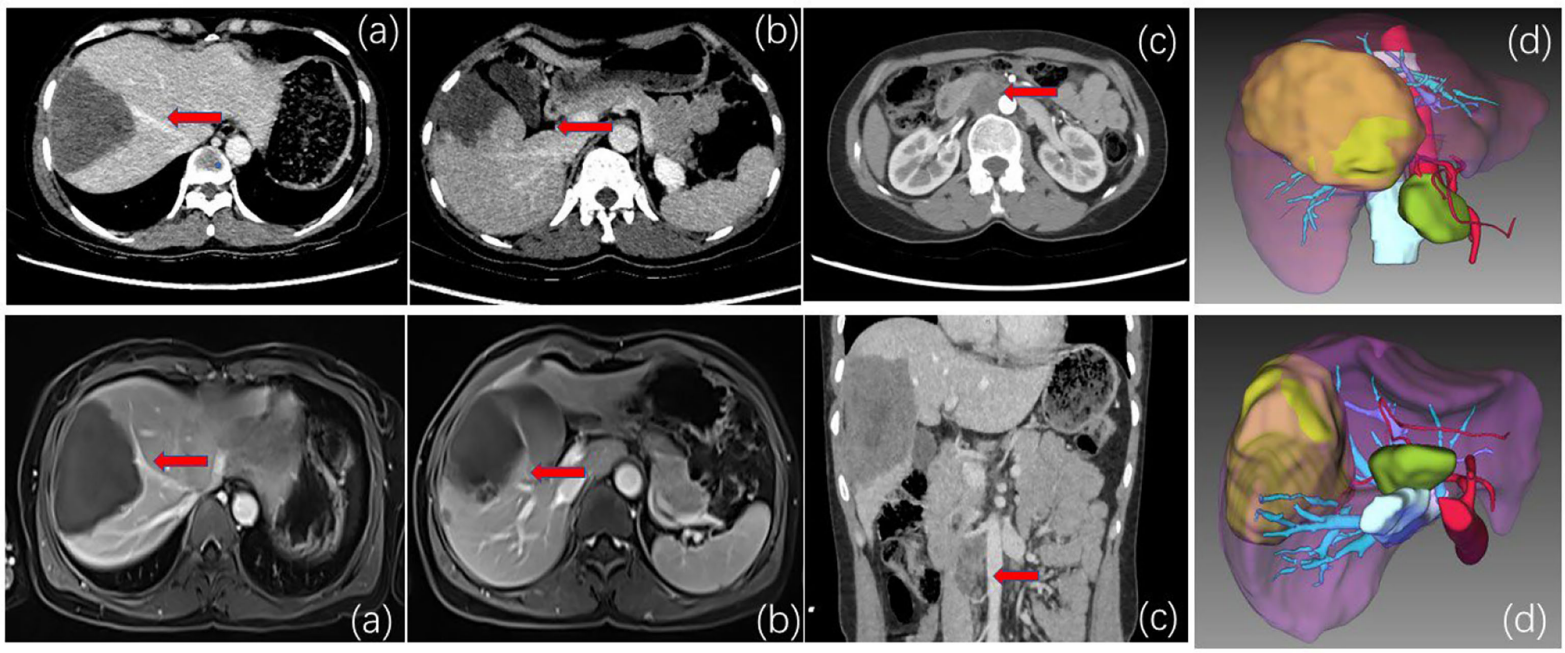
**(A)** CT and MRI venous phase. The red arrow indicates that the middle hepatic vein is close to the lesion and is considered to be invaded. **(B)** CT and MRI delayed phase. The red arrow indicates that the right anterior branch of the portal vein is considered to be invaded. **(C)** CT and MRI. The red arrow indicates inferior vena cava-para-abdominal aortic swelling. Large lymph nodes are approximately 2*2 cm in diameter. The lesions are low signal, and no enhancement is seen after enhancement. Thus, lymph node metastasis of alveolar echinococcosis was considered. **(D)** Preoperative three-dimensional CT and imaging showed enlarged lymph nodes in the inferior vena cava-abdominal aorta. Considered secondary lymph node metastasis of alveolar echinococcosis.

The patient, a 40-year-old female herder living a long nomadic life, was found to have an occupying lesion in the right lobe of the liver on physical examination at a local hospital 2 years ago, with a lesion size of approximately 8.58cm × 5.87 cm in diameter, and hepatic echinococcosis was considered. During multiple re-examinations by colour Doppler ultrasound, CT, and MRI, no enlargement of the lesion was found, and the patient complained of a stable condition and no obvious pain or discomfort, so she refused to undergo surgery and was treated conservatively with oral abendazole (10-15 mg/(kg-d)). Although albendazole is the recommended clinical drug for multilocular echinococcosis, its own limitations cannot be ignored. The plasma concentration of albendazole during oral administration is only (1.86 ± 0.88) mg/L, while the drug concentration in the hydatid lesions is only 1/100 of the plasma concentration, which is far lower than that of killing Echinococcus multilocularis. Therefore, oral albendazole can only inhibit the growth and reproduction of Echinococcus multilocularis, and the direct killing effect is weak. Because albendazole is poorly water-soluble and difficult to be absorbed by the intestinal tract, its bioavailability is low, and long-term medication is likely to cause drug resistance of parasites, and the effect is still poor. This admission was due to “intermittent upper abdominal pain for more than 1 month”. The local People’s Hospital performed abdominal CT scans: a large patch of mixed density was seen in the right lobe of the liver, the largest cross-section was approximately 98 mm*72 mm, and the lesion has grown larger than before and is accompanied by suspected distant lymph node metastasis. After treatment, the pain symptoms were not significantly relieved, and transfer to our hospital for further treatment was recommended. On admission, physical examination showed no yellowing of skin, mucous membrane or sclera and positive pain in the right upper abdomen and under the xiphoid process. Laboratory tests (see **Table 1**). After admission, imaging examination showed no metastasis of alveolar echinococcosis in lung and brain. Abdominal enhancement CT and MRI showed that the liver contained a mixed-signal mass with isosignal in the S7-8-5 segment, with a range of approximately 9.67x7.12 cm, and irregular stripes and lamellar long T2 signal shadow within it. The DWI image of the lesion was low signal, with no enhancement was seen after enhancement. Small nodule-like foci were seen around it, which was mostly considered as a vesicular encapsulated worm, and intrahepatic metastasis was not excluded. The right posterior branch of the portal vein and the middle hepatic vein were found around the lesion and were considered to be invaded (**Figure 1**). Lymph node shadows were approximately 2.0×2.0 cm in size, which is considered stage IV(P3N0M1) (see **Table 2**).

**Table 1:** Laboratory and Imaging examination data on admission.

Biochemical data		Viral markers	
WBC, ×10^9^/L	4.24	HIVAb	Negative
Eosinophils, ×10^9^/L	0.13	HBsAg, mIU/mL	30.6
Basophils, ×10^9^/L	0.03	HCVAb	Negative
Lymphocytes, ×10^9^/L	1.37	HAVAb	Negative
RBC, ×10^12^/L	4.15	HDVAb	Negative
Haemoglobin, g/L	115	HEVAb	Negative
Platelets, ×10^9^/L	346	HGVAb	Negative
PT-INR	0.95	Tumour markers	
PT, S	11.4	AFP, ng/mL	1.65
APTT, S	30.6	CEA, ng/mL	0.8
AlB, g/L	42	CA19-9, U/mL	15.28
Total protein, g/L	72.2	SCC, ng/mL	0.4
AST, U/L	25	CYFRA21-1, ng/mL	1.62
ALT, U/L	28	CA-125, U/mL	23
T-Bil, umol/L	4.3	NSE, ng/mL	12.14
ALP, U/L	61	ProGRP, pg/mL	28.27
γ-GGT, U/L	22	IgG	Positive
AMY, U/L	59	Examination of CT and MRI	
BUN, mmol/L	62	Lesion diameter, cm	9.7 × 8.6 cm
Creatine, mmol/L	45	Lymph node diameter, cm	2 × 2 cm
Natrium, mmol/L	137.2	Child–Pugh	A
Potassium, mmol/L	4.49		
GLU, mmol/L	4.7		
Preoperative ICG	5.80%		

**Table 2:** The patient’s PNM stage grouping of alveolar echinococcosis.

Stage	Category		M
	P	N	
I	1	0	0
II	2	0	0
IIIa	3	0	0
IIIb		1	0
	4	0	0
IV	4	1	0
	any	any	1

## Evaluation

For hepatic alveolar echinococcosis complicated by liquefaction cavitary echinococcosis or cholangitis caused by biliary invasion, it is necessary to intervene in puncture and drainage of the liquefied cavity of the complicated infection, decompress the biliary tract, and control the infection. Fenestration might be more optional. It was feasible to perform first-stage open window decompression (aspiration and decompression of cystic lesions of vesicular worms), and then to perform second-stage radical resection after the healthy side of the liver had been augmented. However, in addition to the large hepatic lesion with suspected metastasis in the distal lymph nodes, considering that the patient had refused previous surgical treatment, and oral abendazole failed to inhibit the aggravation of the derivation of the disease, the open window drainage (palliative therapy) at this time may increase the risk of postoperative bile leakage, infection and recurrent metastasis, or even the loss of the operation due to complications. The best time for surgery was lost due to complications. It is difficult to expose the enlarged retroperitoneal lymph node, which are in close relationship with the inferior vena cava-abdominal aorta and the right kidney during the operation. There is a risk of haemorrhage leading to the patient’s death. However, the treatment of end-stage hepatic vesicular echinococcosis, especially those invading the vena cava or the hilar region, relying only on oral anti-echinococcosis drugs is less effective and cannot achieve the desired therapeutic effect. Therefore, after MDT discussion, it was concluded that although the growth pattern and biological behaviour of hepatic vesicular echinococcosis resembled malignant tumours, it was still a benign disease, and the patient was considered to have indications for surgery. Surgery can achieve the following 2 objectives: (1) radical resection of intrahepatic lesions; (2) removal of suspected metastatic lymph nodes, if the preventive lymph nodes are not removed, parasitic tissue will be left, and thus the surgery would wrongly be judged a cure, which will increase the possibility of postoperative recurrence and multi-organ metastasis. In addition, if surgical intervention is not performed in a timely manner, the patient could develop complications such as lesion invasion of the hepatic hilum, causing obstruction of the hilar vessels and bile ducts and biliary tract infection. In surgical plan 1, both the right posterior branch of the portal vein and middle hepatic vein were invaded, and “right hepatectomy + lymph node dissection” was proposed. In surgical plan 2, if the right posterior branch of the portal vein could be preserved by intraoperative investigation but the middle hepatic vein was invaded, “middle hepatic lobectomy + lymph node dissection” was proposed. The key point and difficulty of the operation was how to remove the suspected metastatic lymph node. Due to the complexity of the disease, the difficulty of laparoscopic surgery and the increased risk of bleeding, open surgery was appropriate. The family agreed to the surgery and signed an informed consent form.

## Surgery Therapy

**Figure 2:**
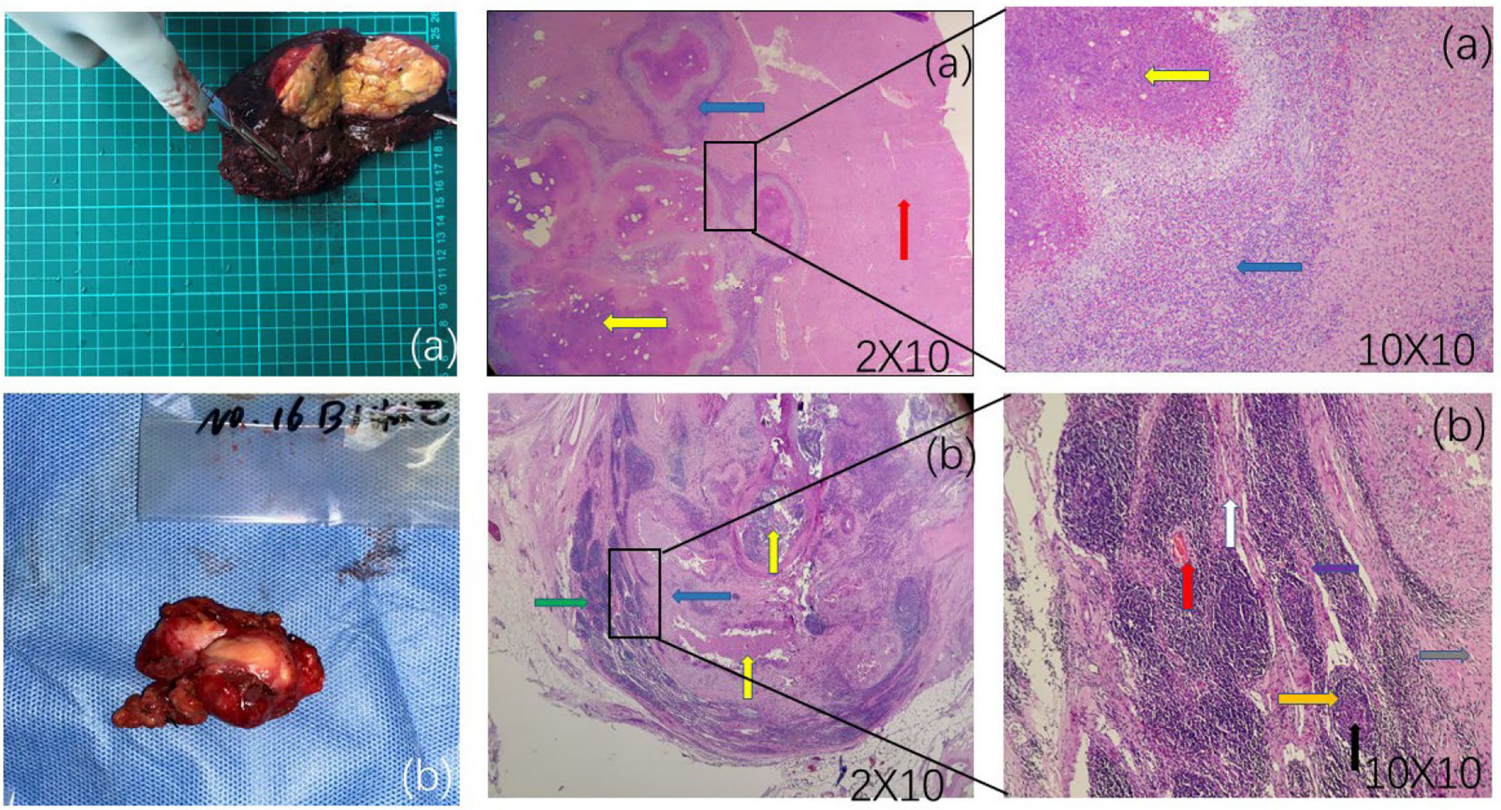
**(A)** A cheese-like or milky-yellow section of the liver specimen with a hard surface. Postoperative liver pathology observed under different magnifications of microscopy: red arrows show normal liver tissue, yellow arrows show hepatic vesicular echinococcosis lesions, and blue arrows show alveolar echinococcosis reaction zones containing inflammatory cells such as eosinophils, lymphocytes and fibroblasts. **(B)** An enlarged lymph node adjacent to the inferior vena cava-abdominal aorta. Postoperative lymph node pathology microscopically observed at different magnifications: yellow arrows show lymph node alveolar echinococcosis lesions, and blue arrows show the reaction zone. A wider reaction zone indicates longer invasion time. More surrounding fibrous tissue is seen, with more fibroblasts and fewer eosinophils, lymphocytes, and epithelioid cells. A narrower the reaction zone indicates shorter invasion time, with fewer corresponding fibroblasts and more eosinophils, lymphocytes, and epithelioid cells. Lymphocytes and epithelial-like cells are increased. Purple arrows show macrophages, red arrows show blood vessels, black arrows show lymph node germinal centres, orange arrows show lymphoid follicles, grey arrows show epithelioid cells, white arrows show fibrous structures of lymph nodes, and green arrows show lymphatic tissue perithelium. Postoperative pathology shows alveolar echinococcosis within the lymphoid tissue.

After anaesthesia took effect, the patient was placed in the supine position, routinely disinfected and towelled. A reverse “L” shaped incision of approximately 20 cm was made in the upper abdomen, and the abdomen was incised layer by layer. The incision towel was laid, and the abdominal cavity was fully exposed. The lesion was located in the middle lobe of the liver, approximately 9*8 cm in size, closely related to the base of the gallbladder, with no obvious adhesions to the surrounding tissues and no metastatic foci in the peritoneum and pelvis. Enlarged lymph nodes were seen next to the hepatoduodenal ligament, behind the head of the pancreas and next to the inferior vena cava. The lymph nodes next to the inferior vena cava were approximately 2*2 cm in size and partially fused with the wall of the inferior vena cava. The distal middle hepatic vein and the right anterior branch of the portal vein penetrated the lesion. The hepatic round ligament, sickle ligament and left triangular ligament were first dissected. Cholecystectomy was performed because of the close relationship between the lesion and the base of the gallbladder. 2. The first hepatic port was preset with a blocking band, and the resection range was marked 1 cm from the lesion. The dorsal membrane of the liver was cut with an electrosurgical knife to cut off the duct system leading to the lesion. The proximal end was ligated to protect the right posterior branch of the portal vein and gradually complete resection of the tumour body (HAE). The ductal system was ligated and sutured, and the wound was closed with continuous sutures of 3-0 proline thread. Hot compresses were applied to the wound, careful haemostasis was achieved, and distilled water was used to fully flush the abdominal cavity to check for active bleeding and bile leakage. 3. The duodenum was fully freed, the stomach and omentum were further pulled upwards, and the common hepatic artery and the proper hepatic artery were found along the gastroduodenal artery. The area was carefully ligated and cut off; otherwise, these small arteries are not taken care of and can be easily damaged and bleed. The lymphatic and adipose tissues of the hepatoduodenal ligament were removed starting from the duodenal bulb, mainly around the hepatic artery. For the enlarged lymph nodes adjacent to the inferior vena cava-abdominal aorta, due to their special location, intraoperative exploration revealed not only partial fusion with the inferior vena cava but also close to the right renal vein, right renal artery, anterior longitudinal ligament of the conus, etc. During the operation, the adjacent tissues were carefully separated, the bleeding points were ligated one by one, and the inferior vena cava, abdominal aorta, right kidney and left renal vein were separated and suspended. Intraoperatively, the lymph node lesion was seen to be partially fused with the inferior vena cava junction. For complete excision of the lymph node, the fused portion of the inferior vena cava and lymph node was excised, and then the inferior vena cava junction was given for repair. During the separation process, the lymph node was still in close contact with the anterior longitudinal ligament of the pyramid. Considering part of the invasion, precise dissection and resection are given to preserve the integrity of the ligament and achieve radical surgery. The surgical field was soaked with distilled water and rinsed. The operation time is about 4.5h, and the intraoperative blood loss is about 50ml. After the operation, the specimen was dissected, the section of the specimen was cheese-like, and the surface was hard. Postoperative histopathological diagnosis: (1) hepatic alveolar echinococcosis; (2) alveolar echinococcosis lesions were seen in the lymphatic tissue, and metastasis was considered (**Figure 2**).

## Postoperative and Follow-Up

After the operation, the patient was given symptomatic treatment with anti-inflammatory agents, liver protection agents, fluid replacement agents, nutritional support and other drugs. On the third day after the operation, chest and abdomen CT showed no obvious fluid accumulation in the operation area, and the drainage tube was removed. ICG 2.4% was detected on the 6th day. There were no obvious abnormalities in routine blood tests, coagulation or liver function. The patient was discharged from the hospital with on the 8th day. He was instructed to take abendazole orally and to regularly review abdominal CT and liver function. No recurrence of hydatid lesions has been found in the telephone follow-up for more than half a year.

## Discussion

The high prevalence of HAE is mainly found in the field of Qinghai Province (8). HAE is characterized as an endemic parasitic disease and is relatively rare, especially with regard to HAE with suspected lymph node metastasis to the inferior vena cava-para-abdominal aorta. There is a lack of clinical consensus on diagnosis and standardized treatment. Lymph node metastasis may be one of the metastatic pathways of HAE, but there is no direct evidence for this, and the prevailing view is now that alveolar echinococcosis destroys the lymphatic vessels in the interstitial tissues of the liver lobules, invades the microlymphatic vessels and then falls off and drains to the local lymph nodes with lymphatic fluid. In 2019, one case of suspected regional lymph node metastasis of hepatic alveolar echinococcosis was treated in our centre (6). From the peripheral sinuses, it proliferates and differentiates to destroy the lymph nodes in the form of spores and eventually forms metastatic lesions of echinococcosis (9). However, there is no histological confirmation of relevant lymph nodes, it is a little inappropriate to conclude “there is distant lymph node metastasis pathway of alveolar echinococcosis”, and further research is needed. Analysis of the source of the enlarged lymph nodes in the inferior vena cava-para-abdominal aorta may consider the following pathways.1. Vesicular echinococcosis invades the right adrenal gland, and the vesicular larvae metastasize to the right hilar lymph nodes, inferior vena cava and lymph nodes around the abdominal aorta. This metastatic pathway is easily overlooked. 2. Lymph node metastasis is in line with the path of hepatic lymphatic drainage. Among the lymph node metastases in the chest and abdomen, the hepatoduodenal ligament is the most common. The metastatic lymph nodes in the portal area easily compress and even erode the portal vein, leading to cavernous degeneration of the portal vein; it can be further to the back of the pancreatic head and metastases to distant lymph nodes around the inferior vena cava and abdominal aorta. 3. Lymph node metastasis of alveolar echinococcosis may also have the characteristics of skipping lymph node metastasis of liver cancer (10), and for these patients we cannot rule out the possibility of skipping lymph nodes directly to the inferior vena cava-preabdominal aortic lymph node under the inferior mesenteric artery. Routine dissection is recommended for regional lymphadenopathy such as hepatoduodenal ligament and hepatic artery. However, there has always been controversy regarding lymph node metastasis around the abdominal aorta. The lymph nodes in NO.16 are closely related to large vessels such as the inferior vena cava, abdominal aorta and renal veins. The surgical risk and trauma are relatively high. If such a wide range of lymph node dissections is performed indiscriminately, the gain will not be worth the loss. Para-aortic lymph node dissection is a more extensive lymph node dissection. Further analysis and research have found that when the cancer metastasizes to the two areas A2 and B1 (11), it is more concentrated in the abdominal aorta. In the lateral arterial group, the anterior group and the abdominal aorta-inferior vena cava group, retroperitoneal lymph node dissection is relatively safe and feasible, and its significance for the thoroughness and long-term efficacy of gastric cancer surgery needs to be further studied. However, there are no clinical guidelines and consensus on the treatment of patients with alveolar echinococcosis with lymph node metastasis. Takahiro AmanoT reported a case of hepatic alveolar echinococcosis with pancreatic head lymph node metastasis in 2018. Endoscopic ultrasonography + FNA was performed before the operation. They concluded that puncture biopsy for alveolar echinococcosis is a contraindication and that there is a risk of allergic reactions or implantation metastases due to spillage of the capsule fluid (12, 13). For patients with alveolar echinococcosis complicated with giant cyst necrosis, external drainage and decompression of the cystic cavity can be performed in phase I, followed by radical resection in phase II after the healthy side of the liver has proliferated. For complex HAE, the echinococcosis lesions should be removed or excised as far as possible, with an area of more than 0.5 cm (14). In addition, for patients considering secondary lymph node metastasis, routine lymph node dissection does not mean a certain cure because pathological tissue may not detect echinococcosis. Urdiales-Viedma reported a case of enlarged lymph nodes in the hilar region in a patient with hepatic cystic echinococcosis. It was characterized by an increased number of follicles and a predominant infiltration of plasma cells, but no parasitic component was seen (15). In 2009, K. Buttenschoen (16) of the United States believed that the surgical method for alveolar hydatid disease should not be limited to the liver but should be extended to routine resection of regional lymph nodes. Routine surgical area lymph node dissection can reduce postoperative hydatid recurrence. In addition, previous animal experimental studies have shown that hepatic alveolar echinococcosis can metastasize to the lymph nodes adjacent to the hepatic artery through lymphatic vessels, but it is not yet certain whether there is hepatic alveolar hydatid disease in humans that metastasizes to distant organs *via* lymph. HAE as an immune-related disease, progresses rapidly in immunodeficient patients. This shows that the body’s immune function has a powerful inhibitory effect on the progression of alveolar hydatid disease. For locally or distantly enlarged lymph nodes, such as obstruction, compression and other space-occupying effects, the suspected metastatic lymph nodes can be removed based on the principle of individualized treatment according to the patient’s condition. Because the inferior vena cava-para-aortic lymph nodes of the patient were large, if it continued to progress, the risk of distant metastasis or haemorrhage caused by compression or vascular invasion could not be ruled out. The pathology of the patient’s hepatoduodenal ligament and the lymph nodes behind the head of the pancreas showed reactive hyperplasia of lymph nodes. The reason may be the stage of reactive hyperplasia of lymph nodes. Alveolar larvae could hardly be detected in pathological slices. Only the pathology of the inferior vena cava-para-aortic lymph nodes was positive, and the lesions showed coagulated necrotic tissue. In the early and middle stages of lymph node metastasis of HAE, focal necrotizing granuloma formation can be seen in pathological sections. After the tissue structure of the lymph node is completely destroyed in the late stage, the centre of the microscope is completely coagulated and necrotic tissue, with scattered small vesicles of vesicles within it and scattered surrounding inflammatory cells such as macrophages, multinucleated giant cells, lymphocytes, etc., which are typical. The morphology of the granuloma changes, but there is the possibility of progressing to echinococcosis. The pathological tissues of this case showed differences between HAE and lymph node echinococcosis by microscopic observation at different magnifications. The characteristics of alveolar hydatid disease invading the liver are as follows: 1.HAE lesions show large areas of necrosis; 2.”narrow/thin” inflammatory response zone (the reason may be that the liver is not an immune organ, and the immune response is not strong.) with few fibroblasts; and 3. no foreign body macrophages (atypical/or no). Characteristics of alveolar hydatid disease invading lymph nodes are as follows: 1. small irregular necrosis; 2. “wide/thick” reaction zone (because the lymph is an immune organ, the immune response is stronger) with many lymphocytes and many fibroblasts, and is hard, (having many fibroblasts and being hard is a pathological manifestation of hydatid invading lymph nodes for a long time); and 3. foreign body macrophages (many and typical). Alveolar echinococcosis is known to spread to the lungs, bones and brain through the blood route, while lymphatic spread seems to be rare. At present, it is mostly believed that germinal cells may play an important role in metastasis because they can proliferate and differentiate into various “echinococcosis cells” and eventually form metastatic lesions of echinococcosis (17). Evidence has shown that mitotically active germinal cells that develop and differentiate into the postoncospherical metacestode stage are present in the tapeworm (18). In summary, it seems possible that a single germinal cell can be washed away by the lymphatic stream, detained in draining lymph nodes and form lymph node metacestodes. These findings suggest that the hepatic lymph nodes of the case presented were metacronously infested in this way.

In this case, multiple enlarged lymph node infections in the abdominal cavity and liver may also be caused by the haematological dissemination of hydatid tapeworms, which means that the liver and lymph nodes are infected at the same time or consecutively. This route means that echinococcosis tapeworms pass from the portal vein through the capillaries of the liver and after all incidental haematogenous influx into a lymph node that exactly drains the liver. Matsuhisa reported that germinal cells might invade intrahepatic veins and can metastasize to other organs (19). In the case presented, this mechanism could not be ruled out, but the likelihood of this event seems to be small. If infections and similar growth rates occur simultaneously in the liver and lymph nodes, the size of the lesions should be approximately the same. However, the involved lymph node measured 2.0cm*2.0 cm, and the liver lesion measured approximately 9.0cm*8.0 cm. Under these assumptions, simultaneous infestation of the liver and a regional lymph node is rather unlikely.

Choosing the right treatment is the key to improving the curative effect of echinococcosis. Radical surgical resection is still the first choice for the treatment of alveolar echinococcosis. For patients with alveolar echinococcosis accompanied by suspicious lymph node metastasis in the inferior vena cava-abdominal aorta, we adopt individualized treatment to achieve the goal of radical cure as much as possible and perfect liver function assessment, such as ICG and three-dimensional reconstruction, before surgery (20). Right anterior lobe and right hepatectomy were planned before the operation. During the operation, the middle hepatic vein was severely invaded, but the right posterior branch of the portal vein could be accurately stripped and preserved. Therefore, it was decided to perform middle hepatectomy, which not only achieved radical resection but also preserved the maximum remaining liver volume. However, swollen lymph nodes adjacent to the inferior vena cava are considered to be caused by alveolar echinococcosis with distant metastasis. IVC is often the most common site of invasion or metastasis in advanced alveolar echinococcosis. When the lesion invades seriously and the backflow is blocked, the vascular repair process during surgery often needs to reconstruct the IVC by means of repair or replacement, so as to ensure its integrity and patency. The individualized invasion of the inferior vena cava is not only the particularity of the operation, but also a test and challenge for the operator. When the scope of the AE lesion is limited and the defect of the IVC wall after resection of the lesion is less than 1/3 of the lumen circumference, the surgery can be performed at the same time. In the case of ensuring sufficient length and tension of the IVC, the self-suture trimming method of the IVC wall can be used. Wentao Wang have studied 71 patients with HAE who had undergone surgical treatment and divided the patients into an *ex vivo* liver resection and autologous transplantation group and an *in vivo* resection group, concluding that combined hepatectomy and IVC reconstruction could be safely performed *in situ* and *ex vivo* (21, 22). Yang X’sresearch shows that in the case of abundant collateral circulation, IVC does not need to be reconstructed (23). The reconstruction method of IVC mainly depends on the degree of lesion invasion. As the most severely invaded vessel, IVC reconstruction is a key factor involved in the success or failure of the entire operation and the prognosis of patients. However, in cases where multiple organs need to be removed, whether the IVC needs to be reconstructed should be carefully considered. The feasibility of these techniques as well as the short- and long-term outcomes should be evaluated, especially when the lesions are large involving the first and second hepatic hilum. Although combined hepatic and IVC resection involves considerable risk, curative surgical resection can result in long-term survival in those with inferior vena cava invasion (24). The patient’s inferior vena cava was partially fused with the suspected metastatic lymph nodes, and thus the enlarged lymph nodes were precisely separated from the surrounding tissues, such as the abdominal aorta, renal vein, renal artery and anterior longitudinal ligament of the conus. The lymph nodes were excised as completely as possible during the operation, the fused portion was excised, and the inferior vena cava was repaired, which reduced the surgical risk and achieved the goal of surgical cure. During the operation, the lymph nodes are removed as completely as possible to remove the fused part, and then inferior vena cava repair is performed. The patient underwent “middle liver resection + enlarged lymphadenectomy + inferior vena cava repair is correct, after partial resection of the lymph node fusion on the lateral wall of the inferior vena cava, continuous suture repair is performed, which reduces the risk of surgery and also achieves the purpose of radical resection of the lesion. But at the same time, we should increase the attention of patients to the disease, improve the level of cultural education, and develop the medical environment in remote areas. As for how to choose the timing of surgery, try to achieve early detection, early diagnosis and early treatment.

## Conclusion

For patients with HAE accompanied by lymph node suspicious metastasis in special locations and rare cases, alveolar echinococcosis that has a greater impact on organ function is treated first, and then antiparasitic treatment is appropriate. Lymph node dissection should be performed as far as possible to achieve radical surgery, which can reduce the risk of postoperative recurrence and metastasis. As for whether the inferior vena cava needs to be removed, reconstructed or repaired, we should follow the patient’s condition to adopt the principle of individualized treatment. In my opinion, it is very important to explore the presence of remote lymph node metastasis of alveolar echinococcosis in order to reduce the recurrence rate. Combining with the published literature and a case in our centre, we suggest that lymph node metastasis may be one of the ways of alveolar echinococcosis metastasis. We need more convincing cases and research. This case still needs long-term clinical verification, hoping to provide a reference for the diagnosis and treatment of similar clinical cases.

## Data Availability Statement

The original contributions presented in the study are included in the article/supplementary material. Further inquiries can be directed to the corresponding author.

## Author Contributions

XX and XQ: Writing - original draft. CG, XQ, HL, ZW, SH, LH and HZ collected clinical data. CG, YZ, HW: Review & editing. XF and HF: Conceptualization, Project administration, Funding acquisition. All authors contributed to the article and approved the submitted version.

## Funding

This study was supported by the Young and Middle-aged Scientific Research Fund Project of the Affiliated Hospital of Qinghai University (No. ASRF-2021-YB-17), The Research Key Laboratory for Echinococcus of Qinghai Province (No. 2020-ZJ-Y01) and Beijing Natural Science Foundation (Z190024). Thanks to FH and FX for funding.

## Conflict of Interest

The authors declare that the research was conducted in the absence of any commercial or financial relationships that could be construed as a potential conflict of interest.

## Publisher’s Note

All claims expressed in this article are solely those of the authors and do not necessarily represent those of their affiliated organizations, or those of the publisher, the editors and the reviewers. Any product that may be evaluated in this article, or claim that may be made by its manufacturer, is not guaranteed or endorsed by the publisher.
